# Birdshot chorioretinopathy in a male patient with facioscapulohumeral muscular dystrophy

**DOI:** 10.1186/s12348-014-0030-z

**Published:** 2015-03-12

**Authors:** Evangelia Papavasileiou, Ann-Marie Lobo

**Affiliations:** Department of Ophthalmology, Harvard Medical School, Massachusetts Eye and Ear Infirmary, Boston, MA USA; Ocular Immunology and Uveitis Service, Massachusetts Eye and Ear Infirmary, 243 Charles Street, Boston, MA 02114 USA

**Keywords:** Birdshot chorioretinopathy, Facioscapulohumeral muscular dystrophy

## Abstract

We report a case of birdshot chorioretinopathy (BSCR) in a patient with facioscapulohumeral muscular dystrophy (FSHD). A 40-year-old male with history of facioscapulohumeral muscular dystrophy with significant facial diplegia and lagophthalmos presents for an evaluation of bilateral choroiditis with vasculitis and optic disc edema. Clinical examination included fundus and autofluorescence photographs, fluorescein angiography, and optical coherence tomography. To our knowledge, this patient represents the first reported case of birdshot chorioretinopathy with facioscapulohumeral muscular dystrophy. Patients with FSHD can present with ocular findings and should be screened with dilated fundus examinations for retinal vascular changes and posterior uveitis.

## Findings

A 40-year-old man with past medical history of facioscapulohumeral muscular dystrophy (FSHD) diagnosed at age 16 reports painless blurred vision in the left eye with nyctalopia. He noticed bilateral worsening of his vision over the past 1 year. Past ocular history was significant for bilateral pterygia and dry eye syndrome.

**Figure 1:**
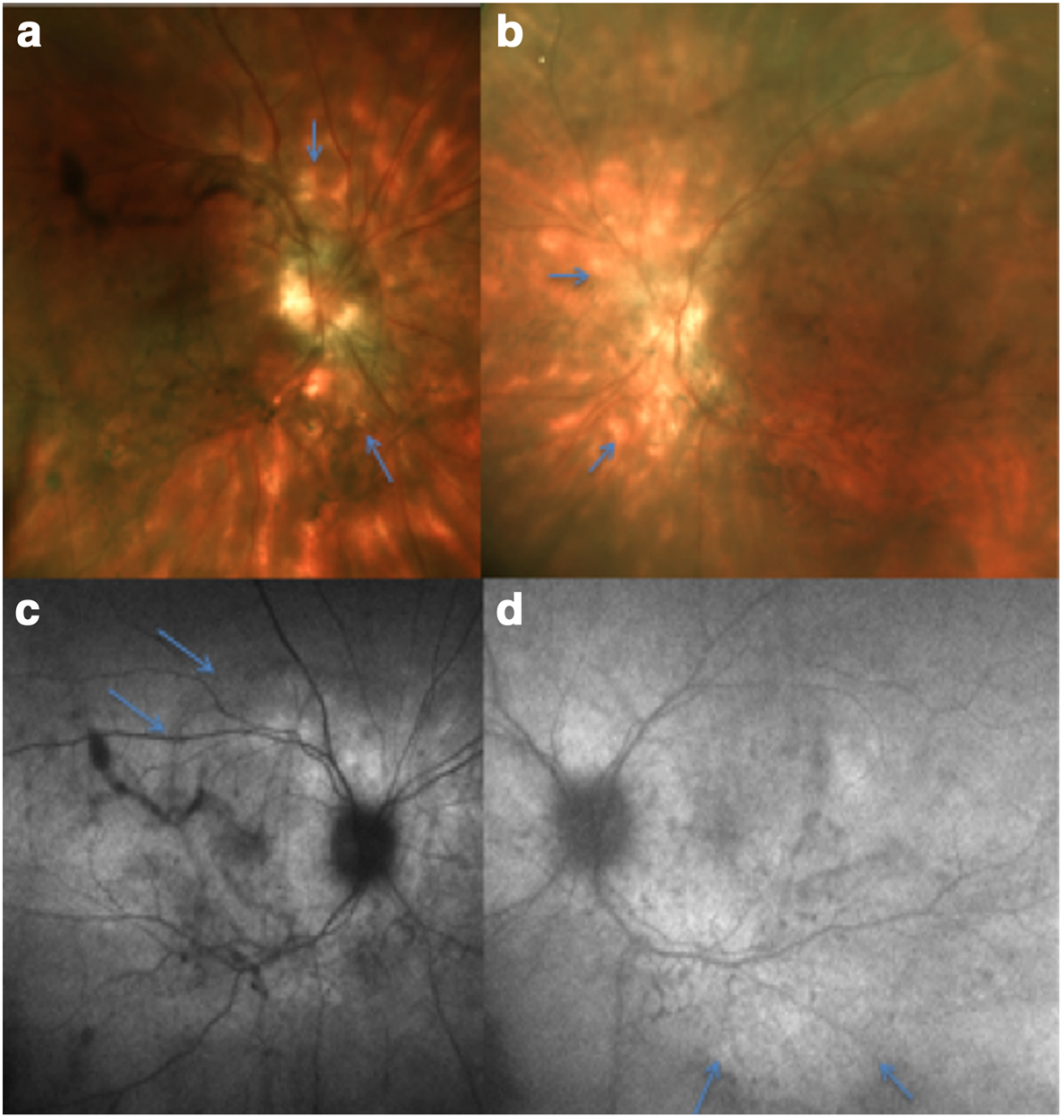
**Color fundus and autofluorescence.** Color fundus wide-field photo of the right eye **(a)** and left eye **(b)** showed multiple cream-colored circumscribed lesions with indistinct borders, most notably nasal to disc (arrows). Fundus autofluorescence photo of the right eye **(c)** and left eye **(d)** showed RPE atrophy at the posterior pole affecting the macula area that did not correspond to the hypopigmented birdshot lesions. The RPE atrophy as suggested by hypoautofluorescence was noted not only in the areas of hypopigmented lesions but also in the areas where there appeared to be no hypopigmented lesions. The arrows show the borders of the abnormal autofluorescence at the posterior pole.

On presentation, visual acuities were 20/30 in both eyes (OU). Intraocular pressures were normal. External examination revealed significant facial diplegia and lagophthalmos. The slit-lamp examination showed bilateral nasal and temporal pterygia. Fundus examination showed 2+ cells in the anterior vitreous and 1+ vitreous haze, vascular sheathing, few scattered pinpoint hemorrhages, and multiple cream-colored circumscribed lesions, most notably nasal to the disc OU (Figure 1a,b).

Visual field testing showed diffuse losses OU. There was hypoautofluorescence with mottling surrounding the peripapillary region and extending along the arcades OU. (Figure 1c,d).

**Figure 2:**
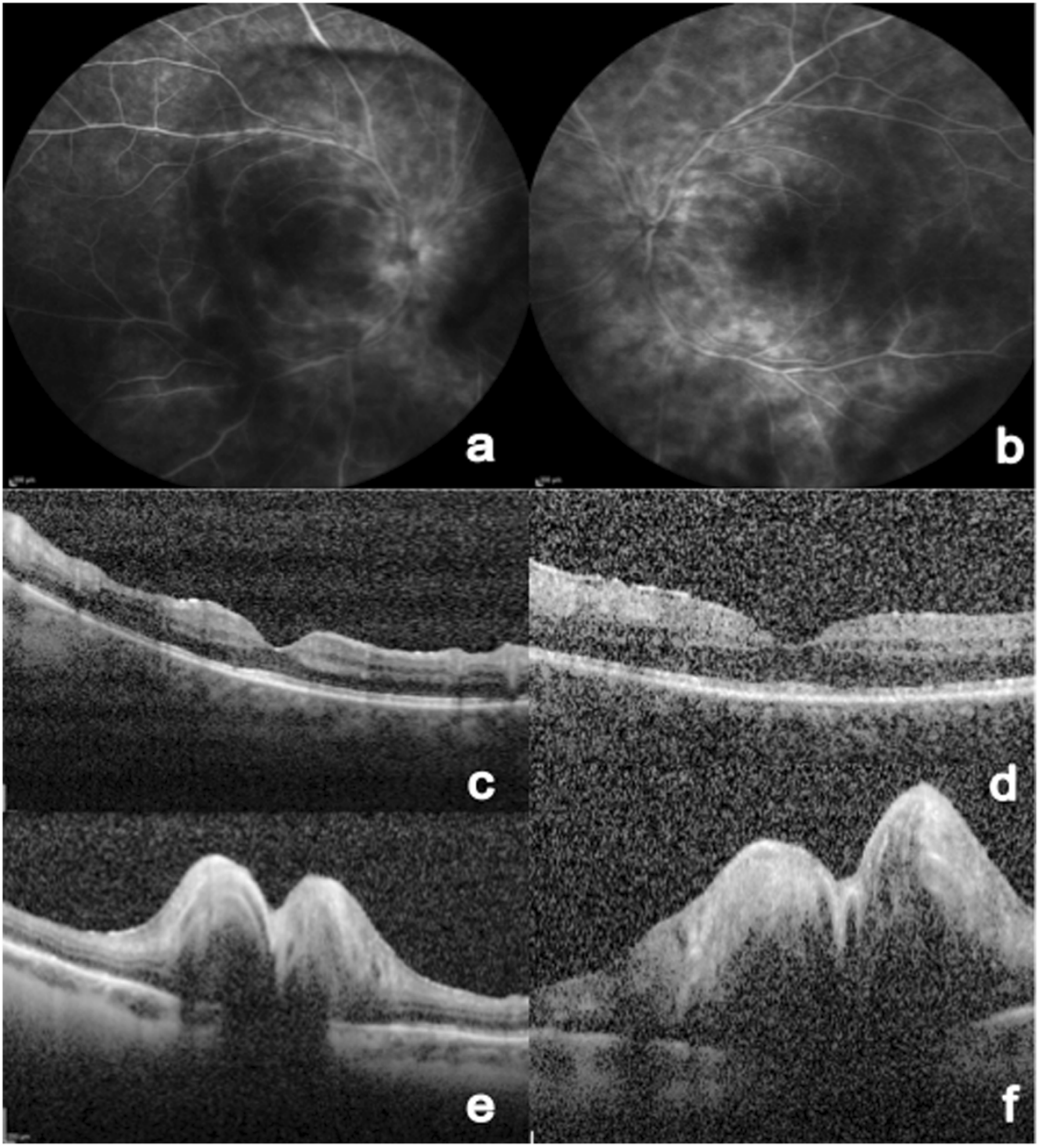
**Fluorescein angiography and OCT image. (a,b)** Fluorescein angiography photo of the right eye demonstrated staining and leakage of fluorescein in the central and peripheral vessels, and staining of the optic nerve head. OCT image at the macula area of the right **(c)** and left **(d)** eye showed no evidence of cystoid macular edema or subretinal fluid at the macula area. There are no changes in the macular architecture, such as loss of the photoreceptor layer. OCT image at the optic nerve head area of the right **(e)** and left **(f)** eye showed elevation of the optic disc.

Fluorescein angiography was notable for diffuse retinal perivascular and optic disc leakage (Figure 2a,b). OCT imaging revealed bilateral optic disc elevation (Figure 2e,f), but no macular edema or subretinal fluid (Figure 2c,d). Full-field electroretinogram testing was normal for the right eye, with the exception of prolonged implicit time of the b-wave maximal response, and abnormal for the left eye, with reduced amplitudes of the isolated and maximal rod responses and a significantly prolonged 30 Hz cone flicker implicit time.

Serologic testing revealed + HLA A-29 and elevated AST with normal ANA titer, Lyme Ab, CBC, ALT, RPR, FTA-Abs, ACE, and lysozyme. Sixty milligrams of prednisone daily was initiated with a slow taper. On follow-up examination, there was improvement in the inflammation with resolution of vitritis, stable optic disc edema, vascular sheathing, and scattered hypopigmented choroidal lesions. Systemic prednisone taper was continued and steroid-sparing therapy with mycophenolate mofetil was initiated.

## Discussion

FSHD is a rare, familial, autosomal dominant myopathy (with a high percentage of sporadic cases) affecting 1 in 20,000 individuals and is characterized by progressive muscle weakness with focal involvement of the facial, shoulder, and upper arm muscles. There is a spectrum of severity in FSHD that appears to be under the influence of several factors. Females are less affected, as are earlier generations of affected family members [1]. Our patient had genetic testing performed which revealed an FSHD deletion mutation of allele 1 (allele size of 25 kb). A larger deletion results in an earlier and more severe disease onset. Extramuscular manifestations of FSHD include hearing loss, retinal findings compatible with Coats disease, and mental retardation [1,2]. Eye involvement includes ptosis, lagophthalmos, abnormal electroretinogram findings in 70% with decreased amplitudes on flicker stimulation and cone stimulation, and a reduction of the b-wave amplitude in the scotopic electroretinogram (ERG) [3] and Coats-like disease [4]. The term retinal telangiectasia has been preferred to Coats disease because of the bilateral findings in FSHD, compared with the unilateral involvement in classic Coats disease [1].

Retinal vascular changes have been described in FSHD and include minor abnormalities such as telangiectasia, aneurysms, capillary nonperfusion, tortuosity of retinal vessels and microaneurysms, and severe cases of intraretinal and subretinal exudative retinopathy that mimics Coats disease in less than 2% [1]. Fitzsimons et al. reported a case series of 75 patients and found that peripheral retinal telangiectasia was found to be extremely common in FSHD muscular dystrophy affecting 50% to 75% of the patients. Of the 75 patients, only 4% showed posterior pole abnormalities and 1% had related visual acuity loss [1]. Massive exudative retinal detachment is a severe but rare manifestation of retinal capillary leakage and telangiectasia [1,5]. There have been reports that patients with bilateral Coats-like disease changes may have an underlying systemic or hereditary disease process, such as FSHD, incontinentia pigmenti, CRB1-associated retinal dystrophy, or familial exudative vitreoretinopathy [6]. Bilateral Coats disease associated with severe FSHD has been reported in an infant [6], three children [5], and an adult female [7]. Most patients have asymptomatic retinal telangiectasia found at ocular screening in adulthood after diagnosis of FSHD.

Birdshot chorioretinopathy (BSCR) is a relatively rare subtype of noninfectious posterior uveitis, which is strongly correlated with the human leukocyte antigen (HLA)-A29 allele even though this is not required for the diagnosis. In our case, the clinical picture was consistent with BSCR and the positive HLA-A29 confirmed our clinical diagnosis. Internationally accepted criteria for the diagnosis of BSCR are based on the presence of bilateral mild intraocular inflammation, ‘birdshot lesions’, and the absence of keratic precipitates and posterior synechiae as seen in our patient [8].

BSCR is a slowly progressive disease with profound dysfunction of vision. Blurring of vision and floaters are the most prevalent presenting complaints [9]. Macular edema occurs in up to 50% of patients with BSCR, and it is the most common cause of visual loss [10]. Our patient complained of nyctalopia and blurred vision. Vitritis and the retinal pigment epithelium (RPE) atrophy at the macula area seen in autofluorescence give a possible explanation for the impaired vision.

ERG abnormalities in patients with BSCR have been well documented. Most common findings include a reduced dim scotopic b-wave amplitude and delayed 30 Hz flicker implicit times in 70% of patients. The dim scotopic b-wave amplitude presumably relates well with night blindness and is a common finding in both BSCR and FSHD [3,11]. Our patient had full-field electroretinogram testing which was abnormal for the left eye, with a significantly prolonged 30 Hz cone flicker implicit time.

This is the first report of BSCR in a patient with muscular dystrophy. There was one reported case of a woman who was diagnosed with congenital muscular dystrophy, bilateral chorioretinitis, optic atrophy, and retinal detachment [12]. There were choroidal atrophy and RPE changes outside the macula area, in contrast with our case where RPE atrophy involved the posterior pole and the macula.

We hypothesize that there might be an underlying immunological nexus between the perivascular inflammation seen in the FSHD muscle and the retinal microvascular abnormalities that also characterize BSCR [13]. T-cells have been implicated in the pathogenesis of both diseases. Increased IL-17 levels have been demonstrated in the aqueous humor of eyes with BSCR [14], and increased serum IL-17, IL-23, and transforming growth factor-beta 1 levels have also been found to be elevated in some treatment-naïve patients with BSCR [15]. In FSHD patients, it was found that inflammatory infiltrates mainly composed by CD8 (+) T cells in muscles and perivascular infiltrates mainly constituted by CD4 (+) cells [16].

The occurrence of BSCR in a patient with FSHD raises the possibility of a common genetic etiology. BSCR is strongly associated with human leukocyte antigen HLA-A29, one of the 21 serologically defined variants of HLA-A gene [9], and FSHD is an autosomal dominant inherited myopathy that is associated in more than 95% of cases with a low number of 3.3-kb tandem repeat units, termed D4Z4, located on chromosome 4q35 [17]. In a recent genome-wide association study, ERAP2 haplotype at chromosome 5q15 was associated with BSCR, suggesting that other genes are involved in the disease process [18]. A recently described transgenic mouse model carrying the D4Z4 array of the FSHD allele developed progressive keratitis of unknown etiology resulting in blindness, supporting the possibility of extramuscular inflammation [19]. Further studies are needed to confirm any overlap of genetic loci in these two rare diseases.

BSCR is considered to be an isolated ocular disorder [20], despite a few reports in the literature describing its possible association with systemic diseases including essential hypertension, cerebrovascular accidents, hearing loss, and cutaneous immune-mediated conditions such as vitiligo and psoriasis [21]. This is the first report of BSCR with FSHD.

Patients with FSHD can present with ocular findings and should be screened with dilated fundus examinations for retinal vascular changes and posterior uveitis.

## Consent

Written informed consent was obtained from the patient for publication of this case report and accompanying images. A copy of the written consent is available for review by the Editor-in-Chief of this journal.

## Abbreviations

BSCR: birdshot chorioretinopathy
FSHD: facioscapulohumeral muscular dystrophy

## References

[1] Fitzsimons RB, Gurwin EB, Bird AC (1987). Retinal vascular abnormalities in facioscapulohumeral muscular dystrophy: a general association with genetic and therapeutic implications. Brain.

[2] Pauleikhoff D, Bornfeld N, Bird AC, Wessing A (1992). Severe visual loss associated with retinal telangiectasis and facioscapulohumeral muscular dystrophy. Graefes Arch Clin Exp Ophthalmol.

[3] Stübgen JP (2007). Facioscapulohumeral muscular dystrophy: multimodal evoked potentials and electroretinogram. Electromyogr Clin Neurophysiol.

[4] Longmuir SQ, Mathews KD, Longmuir RA, Joshi V, Olson RJ, Abràmoff MD (2010). Retinal arterial but not venous tortuosity correlates with facioscapulohumeral muscular dystrophy severity. J AAPOS.

[5] Shields CL, Zahler J, Falk N, Furuta M, Eagle RC, Espinosa LE, Fischer PR, Shields JA (2007). Neovascular glaucoma from advanced Coats disease as the initial manifestation of facioscapulohumeral dystrophy in a 2-year-old child. Arch Ophthalmol.

[6] Ganesh A, Kaliki S, Shields CL (2012). Coats-like retinopathy in an infant with preclinical facioscapulohumeral dystrophy. J AAPOS.

[7] Vance SK, Wald KJ, Sherman J, Freund KB (2011). Subclinical facioscapulohumeral muscular dystrophy masquerading as bilateral Coats disease in a woman. Arch Ophthalmol.

[8] Kiss S, Anzaar F, Foster C (2006). Birdshot retinochoroidopathy. Int Ophthalmol Clin.

[9] Shah KH, Levinson RD, Yu F, Goldhardt R, Gordon LK, Gonzales CR, Heckenlively JR, Kappel PJ, Holland GN (2005). Birdshot chorioretinopathy. Surv Ophthalmol.

[10] Papadia M, Herbort CP (2013). Reappraisal of birdshot retinochoroiditis (BRC): a global approach. Graefes Arch Clin Exp Ophthalmol.

[11] Comander J, Loewenstein J, Sobrin L (2011). Diagnostic testing and disease monitoring in birdshot chorioretinopathy. Semin Ophthalmol.

[12] Avoni P, Monari L, Carelli V, Carcangiu R, Barboni P, Donati C, Badiali L, Baruzzi A, Montagna P (2000). Congenital encephalomyopathy with epilepsy, chorioretinitis, basal ganglia involvement, and muscle minicores. Ann Neurol.

[13] Fitzsimons RB (1994). Facioscapulohumeral dystrophy: the role of inflammation. Lancet.

[14] Kuiper JJ, Mutis T, de Jager W, de Groot-Mijnes JD, Rothova A (2011). Intraocular interleukin-17 and proinflammatory cytokines in HLA-A29-associated birdshot chorioretinopathy. Am J Ophthalmol.

[15] Yang P, Foster CS (2013). Interleukin 21, interleukin 23, and transforming growth factor beta1 in HLA-A29-associated birdshot retinochoroidopathy. Am J Ophthalmol.

[16] Frisullo G, Frusciante R, Nociti V, Tasca G, Renna R, Iorio R, Patanella AK, Iannaccone E, Marti A, Rossi M, Bianco A, Monforte M, Tonali PA, Mirabella M, Batocchi AP, Ricci E (2011). CD8(+) T cells in facioscapulohumeral muscular dystrophy patients with inflammatory features at muscle MRI. J Clin Immunol.

[17] van Deutekom JC, Wijmenga C, van Tienhoven EA, Gruter AM, Hewitt JE, Padberg GW (1993). FSHD associated DNA rearrangements are due to deletions of integral copies of a 3.2 kb tandemly repeated unit. Hum Mol Genet.

[18] Kuiper JJ, Van Setter J, Ripke S, Van T Slot R, Mulder F, Missotten T, Baarsma GS, Francioli LC, Pulit SL, De Kovel CG, Ten Dam-Van Loon N, Den Hollander Al, Huis in het Veld P, Hoynq CB, Cordero-Coma M, Martin J, Llorenc V, Arya B, Thomas D, Bakker SC, Ophoff RA, Rothova A, De Bakker PI, Mutis T, Koeleman BP (2014) A genome-wide association study identifies a functional ERAP2 haplotype associated with birdshot chorioretinopathy. Hum Mol Genet 23(22):6081–6087 10.1093/hmg/ddu307 PMC4204766 24957906

[19] Krom YD, Thijssen PE, Young JM, den Hamer B, Baloq J, Yao Z, Maves L, Snider L, Knopp P, Zammit PS, Rijkers T, van Enqelen BG, Padberq GW, Frants RR, Tawil R, Tapscott SJ, van der Maarel SM (2013) Intrinsic epigenetic regulation of the D4Z4 macrosatellite repeat in a transgenic mouse model for FSHD. PLoS Genet 9(4):e1003415 10.1371/journal.pgen.1003415 PMC3616921 23593020

[20] Pagnoux C, Mahr A, Aouba A, Berezne A, Monnet D, Cohen P, Levinson RD, Brezin AP, Guillevin L (2010) Extraocular manifestations of birdshot chorioretinopathy in 118 French patients. Presse Med 39(5):e97–e102 10.1016/j.lpm.2009.12.005 20219319

[21] Priem HA, Oosterhuis JA (1988). Birdshot chorioretinopathy: clinical charac- teristics and evolution. Br J Ophthalmol.

